# Biophotovoltaics: Green Power Generation From Sunlight and Water

**DOI:** 10.3389/fmicb.2019.00866

**Published:** 2019-04-30

**Authors:** Jenny Tschörtner, Bin Lai, Jens O. Krömer

**Affiliations:** Systems Biotechnology, Department of Solar Materials, Helmholtz Centre for Environmental Research, Leipzig, Germany

**Keywords:** biophotovoltaics, bioelectrochemical system, cyanobacteria, extracellular electron transfer, photo-microbial fuel cell, photosynthesis

## Abstract

Biophotovoltaics is a relatively new discipline in microbial fuel cell research. The basic idea is the conversion of light energy into electrical energy using photosynthetic microorganisms. The microbes will use their photosynthetic apparatus and the incoming light to split the water molecule. The generated protons and electrons are harvested using a bioelectrochemical system. The key challenge is the extraction of electrons from the microbial electron transport chains into a solid-state anode. On the cathode, a corresponding electrochemical counter reaction will consume the protons and electrons, e.g., through the oxygen reduction to water, or hydrogen formation. In this review, we are aiming to summarize the current state of the art and point out some limitations. We put a specific emphasis on cyanobacteria, as these microbes are considered future workhorses for photobiotechnology and are currently the most widely applied microbes in biophotovoltaics research. Current progress in biophotovoltaics is limited by very low current outputs of the devices while a lack of comparability and standardization of the experimental set-up hinders a systematic optimization of the systems. Nevertheless, the fundamental questions of redox homeostasis in photoautotrophs and the potential to directly harvest light energy from a highly efficient photosystem, rather than through oxidation of inefficiently produced biomass are highly relevant aspects of biophotovoltaics.

## Introduction

Humanity faces severe challenges caused by overpopulation and unsustainable lifestyle choices. Even if new energy policies can be efficiently adapted globally, the energy demand by 2040 would still grow by 32% (vs. 2013) to reach 17,934 million tons of oil equivalent (International Energy Agency, 2015). This would require an increased oil production of over 15% and would accordingly increase CO_2_ emissions even further. However, it is predicted that a de-carbonization of the economy might be essential in an effort to curb global warming at 2°C. This requires new energy policies (Schleussner et al., 2016), but more importantly, reducing the usage of fossil fuels in the energy and materials supply chain should be targeted (Mcglade and Ekins, 2015) and the unsustainable waste of resources curbed. While the latter is a responsibility of every individual, the former can only be achieved through the development of novel technologies.

Apart from the energy stored as heat in planet earth, solar power is considered unlimited (based on timeframes relevant for humanity, at least). Green plants and cyanobacteria have developed a natural system that converts water into protons and electrons and in the process produce oxygen as a waste product (Blankenship, 2010). The protons and electrons (also called ‘reducing power’) are then used to reduce the carbon atom in the CO_2_ molecule and thus fix this carbon into multi-carbon units that form the basis for biomass formation and support heterotrophic food-webs. If one calculates the photosynthetic efficiency of such a process based on the light energy hitting the outer atmosphere only less than 1% (theoretical maximum of 4.5%) are actually captured in terms of biomass (Barber, 2009). This means that systems using biomass as a feedstock (not the catalyst) will be significantly less efficient in terms of energy conversion than photovoltaic panels, for instance. However, the photon efficiency of the enzyme complex harboring the water splitting reaction is much higher, with a theoretical maximum of about 70% and, in reality, of about 55% based on the red light spectrum only (e.g., wavelength of 680 nm) (Barber, 2009; Barber and Tran, 2013). The efficiency will decrease to around 20% if counted on the whole solar spectrum, which is still much larger than the biomass-based processes. Thus, the best possibility to utilize the photosynthesis apparatus for energy production is not the production of biomolecules that are later oxidized for energy gain (e.g., biodiesel), but the direct coupling of energy production to the very point of water splitting (photosystem II).

Over the last two decades, a technology using whole cell bacterial catalysts as sources for electrical power has gained a lot of interest (Rabaey and Rozendal, 2010; Harnisch et al., 2015; Kracke et al., 2018). This approach is generally called microbial electrochemical technology, and a system facilitating such a process is termed a bioelectrochemical system (BES) (Schröder et al., 2015). The systems are then grouped depending on the organisms applied and the energy source. For instance, the production of electrical power by parts of or whole phototrophic organisms during illumination is called biophotovoltaics (BPV). The specific feature of a BPV is that it uses natural photosynthesis for direct energy production (Mccormick et al., 2011; Bradley et al., 2012): incoming light is utilized by the oxygenic biomass (e.g., cyanobacteria) to perform the water splitting reaction, and thereby released electrons are subsequently harvested through an anode, i.e., supplying electricity. This principle has only been described in recent years, and while the fundamental electron transfer mechanisms were nicely reviewed a few years ago (Bradley et al., 2012) and several hypotheses based on electron transfer routes reported for heterotrophic electrogens were proposed and discussed (Mccormick et al., 2015; Kaushik et al., 2017), many knowledge gaps remain. Light-to-current efficiency is currently a limiting factor and the BPV system is largely undefined. More recently, Schuergers et al. (2017) discussed the theoretical potential to improve the photocurrent production from the aspect of genetically engineering cyanobacteria.

This review gives an overview on setup configurations and experimental designs used in BPV research during the last decades. We selected key parameters that govern the major characteristics of a BPV system and want to provide a guide for tailoring the appropriate measuring platform with respect to the individual research question. The state-of-art of BPV over recent years is briefly introduced, followed by a systematic summary of the BPV systems.

## State of the Art

There is currently no standardized nomenclature for bioelectrochemical systems that show enhanced current response upon illumination as result of the photosynthetic activity of photoautotrophic organisms. Often it is being termed photosynthetic microbial fuel cell (Pisciotta et al., 2010; Rosenbaum et al., 2010; Kaushik and Goswami, 2018), abbreviated as PMFC or photoMFC. The configurations of PMFCs are quite diverse, no matter how the phototrophs are contacted with the electrode or which type of photosynthetic bacteria is used. Some setups can rely on mediated electron transport by molecules that act as electron shuttles between the biomass and an electrode, but they can also rely on direct electron transfer (DET) facilitated by direct physical contact between (sessile) biomass and the electrode’s surface. In other types of PMFCs, photoautotrophs do not interact with an electrode but serve as oxygen source or as feedstock for heterotrophic exoelectrogenic microorganisms that, in turn, donate electrons to the extracellular electron acceptor. All mentioned types of PMFCs were reviewed and discussed in more detail previously (Rosenbaum et al., 2010).

Biophotovoltaics can be considered as a subcategory of PMFCs, but refers specifically to systems that produce current with sunlight and water (Mccormick et al., 2015). The original electron source is water. Normally, two types of electricity can be detected. Illumination with a light source will activate photosynthesis and therefore the photosynthetic electron transport chain (PETC), which is the basis for the so-called photo currents, photo response or photo power outputs. Removing the light source will lead to a subsequent decrease of the current profile but down to a level still significantly above the abiotic baseline. These are the dark currents or dark voltage levels attributed to the breakdown of endogenously stored carbohydrates. But these stored carbohydrates were synthesized using the electrons and energy generated from the photosynthetic apparatus, and therefore, the dark current should be considered as a ‘delayed’ photo current.

### Electron Transfer Pathways in Cyanobacteria

**FIGURE 1:**
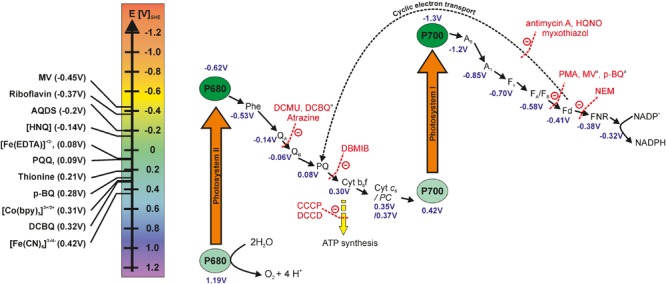
Z-scheme of the photosynthetic electron transport chain, the electron transfer inhibitors at specific sites and potential mediator molecules that could be used for withdrawing electrons. The redox potentials of photosystem I and II subunits are diverse in the literature and the values reported here are obtained from the following sources (Bottin and Lagoutte, 1992; Semenov et al., 2000; Cassan et al., 2005; Allakhverdiev et al., 2010, 2011; Kothe et al., 2013; Caffarri et al., 2014; Schuurmans et al., 2014). The redox potentials of mediators are taken for neutral aqueous conditions from Nivinskas et al. (2002),Schuurmans et al. (2014), Lai et al. (2016), and Emahi et al. (2017). AQDS, 9,10-anthraquinone-2,6-disulfonate; CCCP, carbonyl cyanide *m*-chlorophenylhydrazone; DCMU, 3-(3,4-Dichlorophenyl)-1,1-dimethyl urea; DCBQ, 2,6-Dichloro-1,4-benzoquinone; DBMIB, 2,5-dibromo-3-methyl-6-isopropyl-P-benzoquinone; DCCD, *N-N’*-dicyclohexylcarbodiimide; HNQ, 2-hydroxy-1,4-naphthoquinone; HQNO, 2-heptyl-4-hydroxyquinoline n-oxide; MV, methyl-viologen; NEM, *N*-ethylmaleimide; PMA, phenylmercuric acetate; p-BQ, p-benzoquinone. ^a^DCBQ, MV and p-BQ are performing more as competitors for the natural electron acceptor rather than inhibitors that bind and block the activities of specific sites (Ravenel et al., 1994).

Photoautotrophic cyanobacteria possess the ability to perform oxygenic photosynthesis, a process that converts water and CO_2_ into biomass initiated by incoming light energy. The cellular compartments that harbor all protein complexes and electron mediator molecules involved in the PETC are called thylakoids, which are a partly stacked membrane system located in the cytoplasm. The space inside of the thylakoids is called thylakoid lumen. The protein complexes involved in the PETC are photosystem I and II (PSI and PSII, respectively), the cytochrome *b_6_f* (Cyt *b_6_f*) complex, and the proton gradient-driven ATPsynthase. Electrons are passed from PSII via the Cyt *b_6_f* complex and PSI to the final acceptor NADP^+^, aided by several soluble electron carrier molecules and the ferredoxin NADP oxidoreductase (FNR). The ATPsynthase does not participate in electron transport. But ATP synthesis is fueled by a proton gradient build up across the thylakoid membrane (fed by PSII and the Cyt *b6f* complex). ATP and NADPH that are formed in the so-called *light reactions* of photosynthesis are utilized in the Calvin–Benson–Bassham (CBB) cycle (which is not directly dependent on light and therefore also termed *dark reactions*) where the stepwise conversion of CO_2_ to more reduced states takes place. The Z-scheme (Figure 1) is a schematic display of molecules and protein complexes involved in the PETC, arranged in electron uptake sequence and according to their respective potentials. The left panel in Figure 1 presents some redox molecules (i.e., mediators) that can potentially be used to withdraw electrons from the photoautotrophs at different sites.

Upon incoming light, charge separation occurs within PSII, and electrons are passed to the plastoquinones (PQ) Q_A_ and Q_B_, with the latter molecule leaving PSII and entering the PQ pool. Simultaneously, PSII is re-reduced by receiving an electron released from the water splitting reaction by the oxygen evolving complex. Electrons are further passed from the PQ pool to the Cyt *b_6_f* complex and via the soluble electron carriers plastocyanin (PC) or Cyt *c_6_* to PSI. PSI, too, gets excited by light, passing an electron via ferredoxin (Fd) to the FNR. This enzyme catalyzes the transfer of two electrons and two protons to NADP^+^, forming NADPH + H^+^. In case of a very high NADPH to NADP^+^ ratio and short of ATP supply, electrons may be transferred from Fd to the Cyt *b_6_f* complex, a process called cyclic electron transport around PSI or *cyclic photophosphorylation*. This cyclic electron flow contributes to the proton gradient across the thylakoid membrane and hence ATP production, but no new water molecules are split, so the electrons cannot be harvested for current generation. More detailed descriptions of the PETC can be found elsewhere (Barber and Tran, 2013; Orr and Govindjee, 2013; Mullineaux, 2014).

Not only does the PETC exist in cyanobacteria, but also the respiratory electron transport chain (RETC) is present that maintains the transmembrane proton gradient in the dark by utilizing the hydrocarbons built up in the CBB cycle (Mullineaux, 2014). The RETC locates on both thylakoid and cytoplasmic membrane. Respiration might be regarded as the reverse process of photosynthesis (Vermaas, 2001; Nagarajan and Pakrasi, 2016) for electron and redox balance. Both PETC and RETC share the PQ pool and the Cyt *b_6_f* complex plus the soluble electron carriers PC or Cyt *c_6_*.

### The Exoelectrogenic Nature of Cyanobacteria in Biophotovoltaics

**FIGURE 2:**
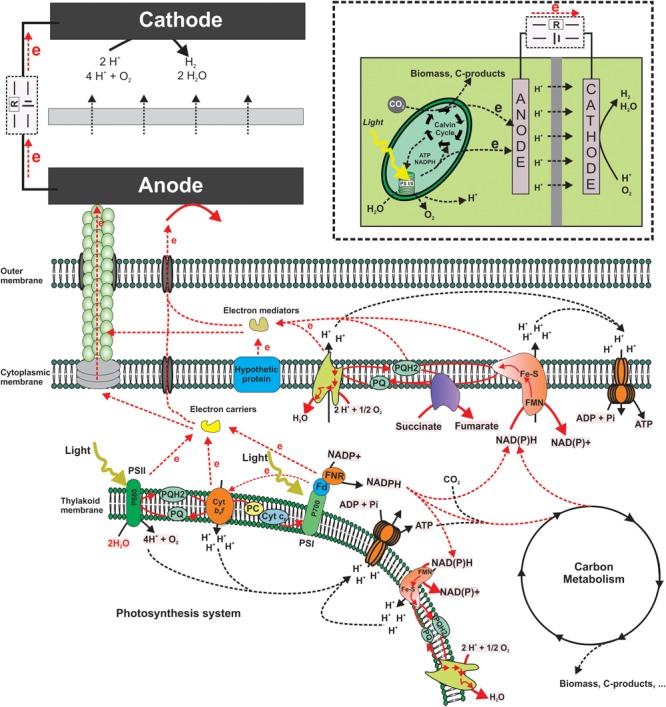
Schematic of biophotovoltaics. Putative electron transfer pathways from the thylakoid membrane components (Photosystems) to an anode. This includes mediated electron transfer as well as hypothetical direct transfer via pili or other membrane structures. Red lines indicate hypothetical electron transfer routes. The insert on the upper right corner shows a schematic structure of a typical BPV system.

While there are diverse reports demonstrating light dependent current output of a BPV, the electron transfer from the photosystem to an electrode is not well understood. Both PETC and RETC as depicted below (Figure 2) are possible sources of electrons at this point based on the observed current response in both light and dark conditions.

When choosing mediators to target PSI and PSII on the one hand, molecules that are able to cross the cytoplasmic and thylakoid membrane are to be preferred (e.g., hydrophobic quinones, etc.). On the other hand, the redox potentials of the chosen molecules have to be considered. Electron transfer only occurs from a more negative to a more positive potential based on the thermodynamic feasibility. While the difference between the two potentials will determine the thermodynamic driving force, the larger the difference the faster the transfer rate. However, in order to specifically target PSI and/or PSII the potentials need to be close enough, otherwise the mediators could also target other parts of the electron transport chain, such as hydrogenase and most likely cytochromes. The inner membrane is most likely impermeable for charged molecules. In this case, it would be necessary to have an intracellular redox shuttle to link the PETC to cytoplasmic membrane components (Lea-Smith et al., 2016), although it was suggested that the thylakoid membrane can physically (unclear if this could also be electrochemically) connect with the cytoplasmic membrane in *Synechocystis* sp. PCC 6803 (Pisareva et al., 2011). Furthermore, Saper et al. (2018) demonstrated that an undefined small molecule (<3 kDa) played a critical role in the current output for *Synechocystis* sp. PCC 6803, suggesting that intracellular redox shuttle targeted on the PETC might be present in cyanobacteria and useable in a BPV. Ferredoxin and NADPH are other possible electron shuttles discussed in the literature (based on inhibitor studies) (Bombelli et al., 2011), as well as flavins and multi-heme cytochromes (based on cyclic voltammetry analyses of anodic biofilms) (Kaushik et al., 2017).

### Cyanobacteria in Biophotovoltaics

Cyanobacterial species tested so far in BPV include *Synechocystis* sp. PCC 6803 (Cereda et al., 2014; Lee and Choi, 2015; Zhang et al., 2018), *Synechococcus* (Tsujimura et al., 2001; Sarma et al., 2018), *Nostoc* (Pisciotta et al., 2010; Sekar et al., 2014; Wenzel et al., 2018), *Arthrospira platensis* (Inglesby et al., 2013), *Anabaena variabilis* M-2 (Tanaka et al., 1985; Tanaka et al., 1988), *Oscillatoria limnetica* (Bombelli et al., 2012), *Leptolyngbia* sp. (Hasan et al., 2014; Çevik et al., 2018), and *Lyngbya* (Pisciotta et al., 2010; Pisciotta et al., 2011). The highest power density reported so far was about 610 mW m^-2^ using *Synechococcus* sp. BDU 140432 (Kaushik et al., 2017). Pisciotta et al. (2010) compared the performance of different wild-type cyanobacterial genera and an undefined phototrophic consortium for their electrogenic activity. The electrogenic yield was highest for the microbial consortium from a freshwater pond, whereas *Synechocystis* sp. PCC 6803 showed a quarter of this activity and still only half of the performance of seven other cyanobacterial pure cultures tested. Another study compared the (photo) power outputs of two alga species as well as *Synechococcus* sp. WH 5701 and *Synechocystis* sp. PCC 6803 (Mccormick et al., 2011). *Synechococcus* showed the best biofilm forming properties on ITO-PET anodes (82% of the initial inoculum attached) and power densities almost two orders of magnitude higher compared to *Synechocystis* that was only loosely bound to the electrode and could be easily washed off. Nevertheless, *Synechocystis* sp. PCC 6803 (hereinafter abbreviated as *Synechocystis*) is still the most prominent cyanobacterium for BPV experiments, maybe for the pragmatic reason that *Synechocystis* is a model organism in photosynthesis research and well characterized with fully sequenced genome and abundant tools for genetic manipulation available. Table 1 summarizes the key milestones in BPV research with cyanobacteria over the last decades.

**Table 1:** Summary of the key milestones of BPV research with *Cyanobacteria* in the past decades.

Growth (conditions, temperature, anode chamber)	Species	Anode: material, area	Electrolyte (anodic)	Mediator	Biomass ^b^ [nmol_Chl_/ml]	Condition	Peak current [μA]	Peak power density [mW m^-2^]	Light intensity ^d^ [μmol_Photons_ m^-2^ s^-1^]	Efficiency ^e^ [%]	Reference
Planktonic Anaerobic with N_2_, 30°C, 20 ml ^a^	*Anabaena variabilis* M-2	Reticulated vitreous carbon *^a^*, 800 cm^2^ *^a^*	50 mM phosphate buffer, pH 8	HNQ, 1 mM	50 mg_CDW_	Fresh cells in light	1000 (400 Ω)	5	(white light 500 W projector lamp)	–	Tanaka et al., 1985
						Cells kept in darkness	500 (400 Ω)	1.3		–	
						Heat inactivated cells	0	0		–	

Planktonic Anaerobic with N_2_, 30°C, 20 ml ^a^	*Anabaena variabilis* M-2	Reticulated vitreous carbon *^a^*, 800 cm^2^ *^a^*	50 mM phosphate buffer, pH 8	HNQ, 1 mM	50 mg_CDW_	Light Light Dark	708 636 400	1.25 1.01 0.4	50 20 0	0.012 0.023 –	Tanaka et al., 1988

Planktonic Anaerobic with N_2_ ± 3% CO_2_, 25 °C, 40 ml	*Synechococcus* sp. (UTEX 2380)	Reticulated vitreous carbon *^a^*, 800 cm^2^ *^a^*	50 mM phosphate buffer, pH 8, 25 g/L NaCl	HNQ, 0.25 mM	11 – 22 (0.1 – 0.2 mg_Chl_/ml)	Light	700 (N_2_) 1100 (N_2_ + CO_2_)	3.1 (N_2_) 7.6 (N_2_ + CO_2_)	140 (650 W projector lamp)	0.010 0.025	Yagishita et al., 1993
						Dark	200	0.3		0.001	

Biofilm Anaerobic + Argon, 25°C, 5 ml	*Synechococcus* sp. PCC 7942	DMBQ-carbon paste electrode, 0.07 cm^2^	50 mM phosphate buffer, pH 6	DMBQ (coated on anode)	0.14 nmol (0.125 μg)	Light	0.7	79.7	150 (xenon lamp)	0.244	Torimura et al., 2001

Planktonic Aerobic, 22°C, 125 ml	*Synechocystis* sp. PCC 6803	Carbon cloth 50 cm^2^	Modified BG11 (lacking citrate)	– HNQ, 1 mM	OD_760_ of 0.6	Light Light	– –	0.2 0.59	2.3 (∼ 100 lux, color temperature 6,500 K)	0.04 0.118	Zou et al., 2009
		Polyaniline coated carbon cloth 50 cm^2^		–		Light	–	0.63		0.126	
				HNQ, 1 mM		Light	–	1.47		0.294	

Biofilm Aerobic, 24°C, 150 ml	*Calothrix* *Pseudanabaena* *Synechococcus* *Ananbaena* *Phormidium* *Nostoc* *Lyngbya* *Spirulina* *Synechocystis* *Leptolyngbya*	Polypyrrole coated carbon cloth 50 cm^2^	F2 for marine cultures Modified DY-V for freshwater cultures	–	Not defined	Light	–	0.807 ± 0.018 0.502 ± 0.024 0.472 ± 0.018 0.453 ± 0.046 0.453 ± 0.046 0.414 ± 0.040 0.396 ± 0.049 0.301 ± 0.274 0.228 ± 0.024 0.155 ± 0.046	1.4 (∼ 100 lux, color cool white fluorescent light)	0.265 ± 0.006 0.165 ± 0.008 0.155 ± 0.006 0.149 ± 0.015 0.149 ± 0.015 0.136 ± 0.013 0.130 ± 0.016 0.099 ± 0.090 0.075 ± 0.008 0.051 ± 0.015	Pisciotta et al., 2010

Biofilm Aerobic, 22 ± 2 °C, 12.56 ml	*Synechocystis* sp. PCC 6803	ITO-PET 12.56 cm^2^	BG11 + 5 mM NaHCO_3_	–	12 (150 nmol_Chl_)	Control Dark Light	0.69 ^c^ 1.46 ^c^ 1.46 ^c^	0.03 0.11 ^c^ 0.114 ± 0.034	46 (10 W m^-2^)	0.0003 0.0011 0.0011 ± 0.0003	Mccormick et al., 2011
	*Synechococcus* *sp.* PCC 7942			–		Control Dark Light	27.6 ^c^ 60.2 ^c^ 116	1.5 ^c^ 4.5 ^c^ 8.5 ± 1		0.015 0.045 0.085 ± 0.001	

Biofilm Aerobic, 23°C, 0.150 ml	*Synechocystis* sp. PCC 6803	ITO-PET 0.8 cm^2^	BG11 with no ferric citrate	K_3_[Fe(CN)_6_], 5 mM	40	Dark	–	0.39 ± 0.05	–	–	Bombelli et al., 2011
						Light		∼0.70	10.58 – 690 (2.3 – 150 W m^-2^, red LED, 625 nm)	0.030 – 0.0005	
				K_3_[Fe(CN)_6_], 5 mM	∼1 ^c^ ∼5 ^c^ 20 ^c^ 40 ^c^ 80 ^c^	Light	–	∼0.44 ^c^ ∼0.55 ^c^ ∼0.54 ^c^ 0.70 ± 0.07 ∼0.95 ^c^	230 (50 W m^-2^, red LED, 625 nm)	0.0009 0.0011 0.0011 0.0014 ± 0.0001 0.0019	
				K_3_[Fe(CN)_6_] 0.5 mM 5 mM 15 mM 25 mM 35 mM 50 mM	40	Dark	–
								∼0.17 ^c^ ∼0.38 ^c^ 0.51 ± 0.04 ∼0.49 ^c^ ∼0.19 ^c^ ∼0 ^c^	230 (50 W m^-2^, red LED, 625 nm)	0.0003 0.0008 0.0010 ± 0.0001 0.0010 0.0004 0	
						Light		∼0.47 ^c^ ∼0.69 ^c^ ∼0.95 ^c^ 1.23 ± 0.05 1.23 ± 0.05 ∼0.30 ^c^		0.0009 0.0014 0.0019 0.033 ± 0.001 0.033 ± 0.001 0.0006	

Biofilm Aerobic, 22 ± 2°C, 20 ml	*Pseudanabaena limnetica*	ITO-PET (20 cm^2^)	BG-11 medium	–	5	Control Dark Light	∼0.16 ^c^ ∼0.38 ^c^ ∼0.79 ^c^	∼0.003 ^c^ 0.007 ± 0.001 0.024 ± 0.004	36.8 (8 W m^-2^)	0 0.0001 ± 0.0000 0.0003 ± 0.0001	Bombelli et al., 2012
		Stainless steel (20 cm^2^)				Control Dark Light	∼0.04 ^c^ ∼0.16 ^c^ ∼0.34 ^c^	∼0 ^c^ 0.001 ± 0.000 0.005 ± 0.001		0 0.00001 ± 0.00000 0.0001 ± 0.0000	
		Polyaniline coated FTO (FTO-PANI) (20 cm^2^)				Control Dark Light	∼0.07 ^c^ ∼0.14 ^c^ ∼0.24 ^c^	∼0.001 ^c^ 0.002 ± 0.001 0.003 ± 0.001		0 0.00002 ± 0.00001 0.00003 ± 0.00001	
		Carbon paper (CP) (20 cm^2^)				Control Dark Light	∼0.03 ^c^ ∼0.06 ^c^ ∼0.15 ^c^	∼0 ^c^ 0.001 ± 0.000 0.002 ± 0.000		0 0.0001 ± 0.0000 0.0003 ± 0.0000	

Planktonic Aerobic, 21 ± 1°C 31.5 ml	*Synechocystis* sp. PCC 6803	ITO-PET (12.56 cm^2^)	BG11	K_3_[Fe(CN)_6_], 1 mM	2.5	Light	4.63 ± 0.59	0.039 ± 0.008	40 (5 W red LED, 625 nm)	0.0004 ± 0.0001	Bradley et al., 2013
	*Synechocystis* sp. PCC 6803 ΔARTO						∼2.48 ^c^	∼0.024 ^c^		∼0.0003 ^c^	
	*Synechocystis* sp. PCC 6803 ΔCOX						∼1.24 ^c^	∼0.010 ^c^		∼0.0001 ^c^	
	*Synechocystis* sp. PCC 6803 ΔCyd						∼3.08 ^c^	∼0.021 ^c^		∼0.0002 ^c^	
	*Synechocystis* sp. PCC 6803 ΔCOX/ARTO						∼1.88 ^c^	∼0.015 ^c^		∼0.0002 ^c^	
	*Synechocystis* sp. PCC 6803 ΔCyd/ARTO						∼8.25 ^c^	0.101 ± 0.016		0.0012 ± 0.0002	
	*Synechocystis* sp. PCC 6803 ΔCOX/Cyd						∼5.06 ^c^	0.103 ± 0.035		0.0012 ± 0.0004	
	*Synechocystis* sp. PCC 6803 ΔCOX/Cyd/ARTO						4.80 ± 0.83	0.175 ± 0.063		0.0020 ± 0.0007	

Biofilm Aerobic, 22 ± 2°C, 0.0004 ml	*Synechocystis* sp. PCC 6803	InBiSn alloy (0.0003 cm^2^)	BG11 + 250 mM NaCl + 5 mM NaHCO_3_	–	100	Control Dark Light	0.087 ^c^ 0.123 ^c^ 0.135 ^c^	189 ± 32 275 ± 20 294 ± 17	200 (warm white LED/42 W m^-2^)	0.435 ± 0.074 0.633 ± 0.046 0.676 ± 0.039	Bombelli et al., 2015

Biofilm, Anaerobic, RT, 100 mL	*Synechococcus* sp. BDU 140432	SF/GE, 3.84 cm^2^	ASN III (artificial seawater) + 750 mg L-1 acetate	–	OD_750_ ∼ 0.4	Light Dark	370 188	220 –	69 (15 W m^-2^, white light)	1.5 –	Kaushik et al., 2017
		SF/QD/GE, 3.84 cm^2^				Light Dark	326 295	–		–	
		SF/QD/GNP/GE, 3.84 cm^2^				Light Dark	483 443	610 –		4.1 –	

Biofilm Aerobic, 20-25 °C, 10 ml	*Synechocystis* sp. PCC 6803	Nanoporous ITO on FTO-glass 1 cm^2^	BG11 + 10 mM phosphate	–	13.4	Light	∼0.95 ^c^	∼3.77 ^c^	512 (LED white light)	∼0.0034 ^c^	Wenzel et al., 2018
		Microporous ITO on FTO-glass 1 cm^2^					∼1.1 ^c^	∼4.37 ^c^		∼0.0039 ^c^	
		Nonporous ITO-PET 1 cm^2^					∼0.003 ^c^	∼0.01^c^		∼0.00001 ^c^	
	*Nostoc punctiforme*	Nanoporous ITO on FTO-glass 1 cm^2^				Light	∼0.90 ^c^	∼3.57 ^c^		∼0.0032 ^c^	
		Nonporous ITO-PET 1 cm^2^					∼0.011 ^c^	∼0.04 ^c^		∼0.00004 ^c^	
	*Synechocystis* sp. PCC 6803	Nanoporous ITO on FTO-glass 1 cm^2^	BG11			Light	∼0.15 ^c^ ∼0.28 ^c^ ∼0.41 ^c^ ∼0.51 ^c^ ∼0.51 ^c^ ∼0.51 ^c^ ∼0.50 ^c^	∼0.60 ^c^ ∼1.11 ^c^ ∼1.63 ^c^ ∼2.02 ^c^ ∼2.02 ^c^ ∼2.02 ^c^ ∼1.99 ^c^	0 128 248 460 650 820 970 (LED white light)	0 ∼0.0040 ^c^ ∼0.0030 ^c^ ∼0.0020 ^c^ ∼0.0014 ^c^ ∼0.0011 ^c^ ∼0.0009 ^c^	
		Microporous ITO on FTO-glass 1 cm^2^					∼0.51 ^c^ ∼0.58 ^c^ ∼0.69 ^c^ ∼0.85 ^c^ ∼0.86 ^c^ ∼0.85 ^c^ ∼0.84 ^c^	∼2.02 ^c^ ∼2.30 ^c^ ∼2.74 ^c^ ∼3.37 ^c^ ∼3.41 ^c^ ∼3.37 ^c^ ∼3.33 ^c^		0 ∼0.0083 ^c^ ∼0.0051 ^c^ ∼0.0034 ^c^ ∼0.0024 ^c^ ∼0.0019 ^c^ ∼0.0016 ^c^	
		Nonporous ITO-PET 1 cm^2^					∼0.02 ^c^ ∼0.04 ^c^ ∼0.1 ^c^ ∼0.12 ^c^ ∼0.12 ^c^ ∼0.11 ^c^ ∼0.10 ^c^	∼0.08 ^c^ ∼0.16 ^c^ ∼0.40 ^c^ ∼0.48 ^c^ ∼0.48 ^c^ ∼0.44 ^c^ ∼0.40 ^c^		0 ∼0.0006 ^c^ ∼0.0007 ^c^ ∼0.0005 ^c^ ∼0.0003 ^c^ ∼0.0002 ^c^ ∼0.0002 ^c^	

Biofilm Anaerobic + Argon, 25°C, –	*Synechocystis* sp. PCC 6803	ITO-FTO-glass 0.75 cm^2^	BG11	–	2.1 ± 0.4 nmol (2.5 ± 0.5 μg_Chl_/cm^-2^)	Light	0.16 ± 0.03 (E_we_ of 0.3V)	0.63 ± 0.12	46 (10 W/m^-2^, LED red light, 685 nm)	0.0063 ± 0.0012	Zhang et al., 2018
				DCBQ, 1 mM			6.68 ± 1.05 (E_we_ of 0.3 V)	26.7 ± 4.2		0.267 ± 0.042	
							11.03 ± 0.38 (E_we_ of 0.5 V)	73.5 ± 2.5		0.735 ± 0.025	

*ARTO, alternative respiratory terminal oxidase; CDW, cell dry weight; InBiSn, Indium Bismuth Selenium; COX, cytochrome *c* oxidase; Cyd, bd-quinol oxidase; DCBQ, 2,6-dichloro-1,4-benzoquinone; DMBQ, 2,6-dimethyl-1,4-benzoquinone; FTO, fluorine tin oxide; GE, graphite electrode; GNP, graphene nanoplatelets; HNQ, 2-Hydroxy-1,4-naphthoquinone; ITO, indium tin oxide; PET, polyethylene terephthalate; QD, CdTe quantum dots; SF, silk-fibroin. *^*a*^*Estimated from Delaney et al. (1984); *^*b*^*Biomass is reported for the value of inoculation with the unit of nmol_*Chl*_/ml unless specified. Molecular weight of Chlorophyll a used during calculations was 893.51 g/mol; *^*c*^*Peak current densities and peak power densities were estimated from the displayed corresponding power figures; power densities were recalculated normalized to the projected surface area of the anode; ^*d*^for light intensity unit conversion, the following coefficients were used unless reported in the literatures: 1 W m^-2^ = 4.6 μmol_photon_/m^-2^/s^-1^ (Sager and Farlane, 1997) and 100 Lux = 2.3 μmol_photon_/m^-2^/s^-1^ (for natural daylight at 6500 K); ^*e*^efficiencies were calculated based on the peak power output (W m^-2^) versus total instant light intensity (W m^-2^).*

## Electrochemistry and Biology in BPV Systems

Over the last decades a diverse range of rector designs has been used in BPV research (see Table 1). This includes single- and dual-chamber systems, double and triple electrode configurations, mediated systems, the use of pure or mixed cultures of phototrophs as well as systems where only thylakoid membranes or isolated photosystems are immobilized on electrodes *in vitro* (Kothe et al., 2013, 2014; Sokol et al., 2018; Zhang et al., 2018). In this section, we summarize the most important works grouped into a technology section focusing on system design and a physiology section focusing on the biological processes.

### Electrochemical Setup

#### Single Chamber vs. Two Chamber Systems

Following the design of traditional MFCs, the first BPV setups consisted of two chambers, each harboring one of the electrodes, separated by a proton permeable membrane (Tanaka et al., 1985, 1988; Yagishita et al., 1993). The disadvantages of applying a proton exchange membrane (PEM) include a higher internal resistance of the device and fouling, degrading or clogging of the membrane in prolonged operation (Saar et al., 2018). However, when electron mediators are applied, it is important to shield the counter electrode to ensure that the molecules are solely interacting with the working electrode and the biomass in the setup. The two-chambered systems also offer the possibility to run the device as a microbial electrolysis cell, where hydrogen gas is produced at the cathode. In the case of photolysis of water, O_2_ is generated by the microorganisms. Having two chambers separates the two gasses in *statu nascendi*, making subsequent use of the hydrogen possible and reducing the explosion risk. A special setup is the device with an air-exposed cathode coated with a PEM at the side that is in contact with the electrolyte (Bradley et al., 2013; Lee and Choi, 2015). Such systems only have one chamber, but the counter electrode reaction (in this case oxygen reduction) happens outside the chamber.

During the last decade, single chamber setups relying on biofilms or microorganisms immobilized at the anode became popular. Immobilization techniques include direct application of thickened biomass at the anode and subsequent drying before use (Cereda et al., 2014; Sekar et al., 2014) as well as fixation with a dialysis membrane that is pulled over the biomass-anode sandwich (Hasan et al., 2014; Çevik et al., 2018). It was argued that biomass firmly settled down on the anode would not come into contact with the cathode, rendering the separation into two chambers unnecessary (Rowden et al., 2018). For this reason, the simpler single chamber devices currently dominate in the literature. However, in contrast to *Geobacter* biofilms (Bond and Lovley, 2003; Reguera et al., 2005), there is no solid experimental evidence whether DET is happening in such systems, or if compounds released to the medium by the cells act as mediators, although Saper et al. (2018) suggested *Synechocystis* could secret some small redox active molecules.

In another setup type, separation of anolyte and catholyte in a microchannel BPV was facilitated using laminar streams of different velocity to create a diffusion-controlled barrier. The authors discuss that fast diffusing species like protons are still able to cross that barrier whereas the charged mediator molecules oxidize at the anode (Saar et al., 2018). Interestingly, even though omitting the PEM, the respective study still presents a system that is separated in two chambers: one chamber acts as *charging unit* where microorganisms are illuminated and transfer electrons to mediator molecules that are subsequently injected into the *power delivery unit* where interaction with the electrodes takes place. In traditional (P)MFCs, charging and power delivery takes place in the same compartment.

#### Electrodes

Power generation in BPV usually relies on the transfer of electrons that are generated within the photosynthetic microorganisms to a working electrode poised as anode. Choosing the appropriate working electrode will have a major impact on current or power outputs in a BPV. The level of material purity has to be considered to minimize unwanted side reactions, e.g., with compounds of the electrolyte (growth medium, buffer etc.). If a biofilm-based system is favored, the electrode material should support attachment and provide a large surface area for growth. Because of comparably very low current outputs, plain carbon anode materials like those found in BES research are rather scarce in BPV systems relying on cyanobacteria as whole cell catalysts. Therefore, surface modifications or coating of the electrode base material known from bioelectrochemical literature have found their way into BPV research. For instance, indium tin oxide (ITO) is a popular coating agent for anodes in BPV devices. The sheet resistance of ITO layers is dependent on the thickness of the layer and decreases with increasing layer thickness. Bombelli et al. (2012) compared the power outputs of *Pseudanabaena limnetica* biofilms on ITO-PET, stainless steel, carbon paper and conductive polyaniline coated on fluorine doped tin oxide (FTO) coated glass. Biofilms grown on ITO-PET and stainless steel electrodes performed best in terms of photo response and light to dark power output ratios whereas carbon paper was found to be rather unsuitable. The authors discussed that the surface energy of the material has even greater impact than the roughness of the surface. Wenzel et al. (2018) compared the performance of *Synechocystis* with three types of ITO-coated anodes that differed in surface porosity. Non-porous ITO on PET was clearly outperformed by FTO coated glass anodes coated with a nano-porous ITO nanoparticle film, or with microporous ITO particles on a nano-porous ITO nanoparticle film that both showed a 300-fold increase in current outputs. Zou et al. (2009) coated carbon paint and carbon cloth electrodes with polyaniline or polypyrrole and observed in both cases enhanced light responses and stronger biofilm formation on electrodes modified with the conductive polymers. In contrast, planktonic *Synechocystis* (without the addition of an exogenous mediator) did not display any improvement of performance regardless of the electrodes coating. For the same BPV setup, changing the anode material from polypyrrole to nanostructured polypyrrole caused a 4.5-fold increased power output from an undefined phototrophic pond consortium (Zou et al., 2010). Another study investigated poly(amidoamine) dendrimers with a ferrocene core as coating agent to optimize electrochemical communication between microorganisms and electrodes (Çevik et al., 2018). Compared to the bare graphite electrode, coating with a second-generation polymer let to an almost 40-fold increase in current output from an undefined phototrophic pond culture, whereas first- and third-generation dendrimers still showed a 10-fold improvement.

The use of electron-conducting redox hydrogels is another attempt to improve the electrochemical communication between microorganisms and electrodes. Hasan et al. (2014) compared the performance of immobilized *Leptolyngbia* on four graphite electrodes coated with different hydrogels from cationic osmium redox polymers. The modified electrodes displayed individual redox potentials, and [Os(2,2′-bipyridine)_2_(poly-vinylimidazole)_10_Cl]^2+/+^ with a formal redox potential of 220 mV (vs. Ag/AgCl/KCl_3M_) was found to promote the thermodynamic driving force for electron transfer best under the chosen experimental conditions. In case of biofilm-based setups, proper and fast attachment of the biomass to the electrode is a crucial step in process design. In a pre-study, Kaushik et al. (2016) were screening biofilm growth of *Synechococcus* sp. BDU 140432 on the biopolymers chitosan and silk, inspired by studies on tissue engineering, in comparison to the synthetic polymers polyaniline, osmium and anionic Nafion. Densest biofilm formation was observed for silk and a silk:chitonsan-blend with more than 31 and 38% increase of cell concentration compared to the blank. These findings were translated to a subsequent BPV study (Kaushik et al., 2017), where silk fibroin was used as supportive coating of the graphite working electrode to promote cell attachment. Following the idea of Förster resonance energy transfer (FRET), performance was further optimized by incorporating cadmium telluride quantum dots and graphene nanoplatelets into the matrix which led to a 50.8-fold increase in power density compared to the bare graphite material. In the respective study, the highest power density reported so far for BPV setups was achieved, reaching 610 mW m^-2^ and a light-to-power efficiency of 4% (Kaushik et al., 2017).

In contrast to the huge variety in BPV anode materials, in case of the cathode, platinum is the most prominent catalyst of choice (Tanaka et al., 1988; Yagishita et al., 1993; Cereda et al., 2014; Gonzalez-Aravena et al., 2018; Zhang et al., 2018) to facilitate the reduction of oxygen and formation of water (see Figure 2). Due to its high oxidation potential and uncritical product formation, oxygen is the most prominent electron acceptor for the cathodic reaction (Logan et al., 2006). Furthermore, as already stated above, in systems physically separated into an anodic and a cathodic chamber, hydrogen formation from protons can also be targeted at the cathode (Pinhassi et al., 2016; Sokol et al., 2018).

#### Electrolyte

The ideal electrolyte for a BPV should provide sufficient conductivity for ion exchange between electrodes and low internal resistances, support cell viability and maintain a stable environment for the desired (electrochemical) reaction(s). Stabilizing an alkaline pH will reduce the energy cost of CO_2_ concentrating process for cyanobacteria and improve the build-up of biomass (Mangan et al., 2016).

In terms of overall conductivity, salt-tolerant or marine species have a clear advantage over freshwater species when the growth medium also serves as the electrolyte. *Synechococcus* biofilms maintained in a 1:1 mixture of BG11 and artificial seawater (conductivity of 43.1 mS) showed power outputs almost two orders of magnitude higher than *Synechocystis* in BG11 (conductivity of 2.5 mS) (Mccormick et al., 2011).

#### Mediators

Redox mediators are molecules that repeatedly take up and release electrons and can therefore act as an electron shuttle between a cell and an electrode. These mediators can be added externally, but can also be produced by the microbes themselves (Rabaey et al., 2005; Marsili et al., 2008). While in principle mediators are necessary for planktonic systems, in biofilm-based setups they might only play a minor role (Zou et al., 2009). Omitting the use of mediators might be advantageous in order to safe costs, avoid possible toxic effects or unwanted side reactions, and will reduce the dependence of the BPV system from the exogenous supply of reactants in the case of artificially added mediators. To the best of our knowledge, there is to date no experimental evidence of DET in phototrophs. In addition, no significant similarities could be found with BLAST of the outer membrane cytochrome complex MtrCAB from *Shewanella oneidensis* (Pirbadian et al., 2014) and omcS or piliA from *Geobacter sulfurreducens* (Reguera et al., 2005; Shrestha et al., 2013) against the cyanobacteria genome in the NCBI database. One needs to keep in mind that the observed current outputs are orders of magnitude smaller than those observed for the above mentioned electrogenic heterotrophs. The very small currents observed in BPVs could even in biofilms be likely attributed to secreted metabolites or cellular components released during cell lysis (Saper et al., 2018).

An advantage of using externally added mediators is that it is possible to target specific points in the electron transfer chain based on redox potentials and in addition they can offer the possibility to reduce the dependence on the electrode surface area for contacting the cells. When choosing mediators, one needs to carefully consider the mid-point potential of the mediator and the point of access to the ETC (Figure 1). It is also advisable to study a range of mediator concentrations in combination with biomass concentrations to find the optimal balance between both parameters. A too low concentration of the mediator could be a limiting factor for the overall current output of a BPV device, whereas a too high concentration might have inhibitory or toxic effects on microorganisms (Bombelli et al., 2011).

The most prominent mediators used in BPV so far are potassium ferricyanide and quinones such as 2-hydroxy-1,4-naphthoquinone (HNQ) (Tanaka et al., 1985, 1988; Yagishita et al., 1993; Zou et al., 2009) and 1,4-benzoquinone (Torimura et al., 2001; Sekar et al., 2014). Bombelli and coworkers used potassium ferricyanide in a two-chamber, two-electrode setup and compared performances between isolated thylakoids from spinach (*Spinacia oleracea*) with *Synechocystis* (Bombelli et al., 2011) studying various Chlorophyll *a* and ferricyanide concentration ratios (see Table 1).

#### Normalization

In BPV, biomass for the inoculation of a setup is often quantified in terms of the Chlorophyll *a* content (Bombelli et al., 2011, 2015; Bradley et al., 2013; Wenzel et al., 2018; Zhang et al., 2018). This parameter is, however, sensitive to environmental conditions and changes dynamically over the lifetime of the biomass. Thus, normalizing the system behavior (e.g., electron output, product formation, etc.) to biomass is probably the better way to evaluate the performance of electrogens in BES and can lay the basis for system optimization. Reporting current density to projected surface area and product formation in volumetric units both ignore the active biomass content involved in the bioelectrochemical process, and therefore cannot distinguish the effects between advanced system design and changed activity of the microorganisms.

### Physiology of Cyanobacteria in Biophotovoltaics

Electrochemical methods, e.g., cyclic voltammetry, chronoamperometry and stepwise polarization, are routinely applied in BPV research (Wenzel et al., 2018; Zhang et al., 2018) and can provide profound information on the redox reactions occurring on the electrodes. However, information about current/voltage is a sum-signal of many biological as well as electrochemical processes and, hence, is not specific enough to describe the metabolic phenotype. The physiology of cyanobacteria under the condition of an external electron sink from an anode is largely unknown to date but it is in our opinion the fundamental basis for understanding and further optimizing the BPV system.

Naturally, cyanobacteria use a complex and dynamic regulation system to balance the reducing power generated by the photosystems (Kramer and Evans, 2011), such as carbon assimilation, storage metabolism and the cyclic electron transfer around PSI. These dynamic systems help the cells to balance their intracellular redox status in the dynamically changing natural conditions. Introducing a new electron sink will force the cells to develop a new equilibrium among all the electron transport routes. What the changes are and whether these changes can benefit the cell’s physiological status is largely unknown. More importantly, rationally engineering the new equilibrium to benefit the electron transfer toward the anode without impairing the long-term fitness and photosynthetic activity is not feasible without quantitative knowledge of the physiological state of the microorganisms in the BPV system. Introducing an additional electron sink could impact the photosystems and/or carbon assimilation. There is a positive correlation between light and photocurrent which points to a connection between photosystems and the anode via unknown intracellular electron carriers. But at the same time, carbon metabolism seems to make an important contribution as well, as demonstrated by the significant currents detected in the dark (see Table 1). A fundamental understanding of these processes will be crucial for the design of efficient BPV systems, because as stressed above, only harvesting electrons from the photosystems directly promises high photon efficiencies. In this section we summarize and critically address the current knowledge about the most important physiological processes in a BPV.

#### Light Source and Intensity

Future application of BPV systems will obviously rely on natural sunlight of varying intensity, changing dark-light cycles and incident angles. During research, we rely on artificial light sources to have the possibility of calculating balances and efficiencies and the knowledge generated might guide future implementation of the technology. When choosing an artificial light source for a BPV one needs to consider the absorption maxima of the light harvesting pigments in the reaction centers of the photosystems and the phycobilisomes (PBS). The PBS are part of the light harvesting complexes and channel photons toward PSII. The PBS consist of phycocyanin and allophycocyanin with an absorption maximum at 630 and 650 nm, respectively. Phycoerythrin (570 nm) is another phycobilin but absent in most cyanobacteria (Govindjee and Shevela, 2011). Chlorophyll *a* absorbs at 440 and 680 nm. Maximum current or power outputs at the appropriate wavelength were demonstrated in several studies (Pisciotta et al., 2010; Mccormick et al., 2011; Sokol et al., 2018). Because light is a necessity in BPV studies, care must be taken on possible abiotic side effects of illumination. This includes warming up of the setup due to intense illumination but also material instability, e.g., decomposition of the mediator potassium ferricyanide under ultra-violet light (Ašpergěr, 1952).

Another parameter to consider is the intensity of the light source. The available light should not become the limiting factor for photo current generation, but too high light intensities will lower the efficiency of the device and can lead to photo damage within the microorganisms. Some studies demonstrate exponential current enhancement with increasing light intensity up to a saturation point (Zou et al., 2009; Pisciotta et al., 2011; Wenzel et al., 2018). Pisciotta et al. (2011) measured levels of dissolved oxygen and photo electrogenic response while increasing the illumination with either red or blue LED light (_max_ = 642 and _max_ = 463 nm, respectively), demonstrating photo inhibition on *Nostoc* biofilms especially by high blue light levels (3,000 lux and above). Altering the light intensity between 2.3 and 150 W m^-2^ had minor effects on peak power outputs of *Synechocystis* (40 nmol Chl mL^-1^ and 5 mM potassium ferricyanide) when an array of red LEDs with an emission peak at 625 nm was used. Though, up to 100 W m^-2^, power increase with increasing light intensity was observed before saturating (Bombelli et al., 2011).

#### Photosystems

The photosystems absorb light to energize electrons and then channel them into a photochemical quenching route and non-photochemical quenching processes (e.g., dissipation as heat or emission) (Campbell et al., 1998; Maxwell and Johnson, 2000; Jakob et al., 2007). Balancing the electron flow along the electron transport chain can quantitatively reveal the photosynthetic efficiencies distributed between dissipation, respiration, alternative electron transfer (including the anode in BPVs) and biomass formation (Jakob et al., 2007; Ruban, 2017). This, however, requires the determination of many parameters such as biomass composition, chlorophyll quantity and its corresponding absorbance/fluorescence spectrum, oxygen evolution rate and respiratory quotient, etc. Detailed descriptions of the methods and equations can be found in literature (Hofstraat et al., 1994; Gilbert et al., 2000; Wagner et al., 2006; Jakob et al., 2007).

Several key parameters are discussed here: Chlorophyll *a* (Chl *a*) is the core catalytic center of PSI and PSII, responsible for capturing the light energy to drive the electron flows. The chlorophyll content can be measured using spectrophotometer at 680 and 750 nm respectively (Zavřel et al., 2015b). In addition to the quantity, the specific absorbance of Chl *a* can also be measured by a spectrophotometer, and this value together with the quantity can then be used to determine the radiation absorbance (Q_phar_ usually given in μmol_photons_/mg_Chl a_/d) by the whole cell under a given light source condition. This gives the quantity of total photons available for the photosystem. Furthermore, fluorescence emission of the chlorophyll at light/dark conditions can be measured by a pulse amplitude modulation (PAM) fluorometer to determine quantum efficiency of PSII and subsequently the fluorescence-based photosynthesis rate (P_F_) if oxygen evolution rate can be determined as well (quantum efficiency of PSII, Φ_PSII_, %; fluorescence/oxygen-based photosynthetic rate, P_F_/P_O_, μmol/mg_Chl a_/d). This gives the maximum amount of electrons channeled into the photosystem (Jakob et al., 2007). However, it needs to be noted that the PAM-based method is based on some assumptions and largely affected by the experimental conditions (Schuurmans et al., 2015; Ruban, 2017).

The rate of oxygen evolution from photoautotrophic bacteria is a direct measure of the catalytic water splitting activity of PSII (Schuurmans et al., 2015). It has been used as an important parameter to assess the effects of chosen BPV experimental conditions, such as addition of inhibitors (Bombelli et al., 2011) or insertion of mutations (Bradley et al., 2013) on the electron transport chains. A conventional plugged-in oxygen probe or Clark-type electrode can be used to measure the oxygen level *in situ* (Zou et al., 2009; Pisciotta et al., 2010) or *ex situ* (Mccormick et al., 2011; Bradley et al., 2013). By assessing the concentration at different conditions, from light to dark or anaerobic to aerobic, the net oxygen evolution rate can be determined and used to address the oxygen-based photosynthetic electron transfer rate (P_O_) (Gilbert et al., 2000). This net rate is biased by alternative electron pathways such as the oxygen consuming processes and electron cycling around PSII (Jakob et al., 2007). Ideally, an off-gas analysis of the BPV would be performed, to determine the net oxygen evolution rate.

#### Study of the Electron Transfer Mechanisms

The electron transfer mechanism is still unclear for BPV as discussed above. One straightforward approach for this study would be creating knock-out/overexpression mutants at specific sites. For studying the involvement of the respiratory terminal oxidases in electrogenic activity of cyanobacteria, Bradley and coworkers created mutants of *Synechocystis* lacking the three enzyme complexes in all possible combinations (Bradley et al., 2013) and compared their potassium ferricyanide reducing capability with the wild-type. The mutants were created using two subsequent homologous recombination steps, and mutant genotypes were confirmed via PCR. The best performance in terms of power density was observed for the triple knockout of cytochrome *c* oxidase (COX), cytochrome bd-quinol oxidase (Cyd), and alternative respiratory terminal oxidase (ARTO) (Table 1).

While genetic manipulation in cyanobacteria is so far still more challenging compared to heterotrophs (Berla et al., 2013), another possibility to decipher electron transfer processes is the use of inhibitor molecules that target specific proteins in the electron transfer chains. Since these might help to find the key steps involved in electron transfer to an anode, we summarize these molecules here:

##### 2-Hydroxy-1,4-naphthoquinone

2-Hydroxy-1,4-naphthoquinone (HNQ) receives electrons between Q_B_ and PSI, targeting several sites of both PETC and RETC (Tanaka et al., 1988; Pisciotta et al., 2010). Solely applied, this synthetic quinone is not suitable to target specific sites of intracellular electron transfer but was used as electron mediator before. This is important: In a BPV, HNQ will act as an electron shuttle.

##### 3-(3,4-Dichlorophenyl)-1,1-dimethyl urea

3-(3,4-Dichlorophenyl)-1,1-dimethyl urea (DCMU) is a PSII-inhibitor frequently used in BPV studies to verify the water splitting reaction as source of photo current electrons (Tanaka et al., 1988; Pisciotta et al., 2010; Pisciotta et al., 2011; Bombelli et al., 2015). DCMU interrupts electron transfer between Q_A_ and Q_B_, hindering linear electron transfer from PSII into the PQ pool. Bombelli et al. (2011) investigated the effects of DCMU on oxygen evolution and power outputs of *Synechocystis*. Interestingly, photo power outputs were decreased by only 63 ± 17% when 15 μM of the inhibitor were added to a cell suspension containing 40 nmol Chl mL^-1^, whereas oxygen evolution was almost completely eliminated. From this, the authors conclude that residual photo power output is not a cause of incomplete inhibition but rather an effect of enhanced electron donation from the RETC. In contrast to this, Sekar et al. (2014) point out that incomplete inhibition is a result of DCMU binding to the Q_A_ site and subsequent slowdown of electron transfer, whereas binding to the Q_B_ site leads to complete inhibition. In another study, biofilms of *Lyngbya sp.* and *Nostoc sp.* where completely and irreversibly inhibited by addition of 25 μM DCMU (Pisciotta et al., 2011). Forty mM DCMU had only minor on planktonic *Anabaena variabilis* M-2 (and HNQ as mediator) in the dark but decreased current outputs under illumination by 20–50% (Tanaka et al., 1988).

##### Atrazine

Similar to DCMU, atrazine binds highly specific to the Q_B_ binding pocket in PSII, blocking (re-)reduction of PQ from the PQ pool. It was used in a study with *Lyngbya sp.* and *Nostoc sp.* biofilms to verify the complete inhibitory effect on photo power output that is caused when disrupting the PETC at this point (Pisciotta et al., 2011).

##### 2,5-Dibromo-3-methyl-6-isopropylbenzoquinone

2,5-Dibromo-3-methyl-6-isopropylbenzoquinone (DBMIB) is known to block electron transfer between the PQ pool and the Cyt *b_6_f* complex by binding to the quinol oxidation site on Cyt *b_6_f* with high affinity. But it also acts as an electron mediator (Yagishita et al., 1993) that can take up electrons from the PQ pool, and was shown to enhance both dark and photo power outputs from different cyanobacterial species (Yagishita et al., 1993; Pisciotta et al., 2010, 2011; Bombelli et al., 2015). The agent is therefore not suitable for inhibitor studies to investigate cellular electron donation sites.

##### Carbonyl cyanide *m*-chlorophenylhydrazone

Carbonyl cyanide *m*-chlorophenylhydrazone (CCCP) affects electron transfer at the reducing side of PSII (Yagishita et al., 1993) but in a relatively non-specific manner compared to DCMU or atrazine (Pisciotta et al., 2011). It is also a proton uncoupler affecting ATP synthesis (Pisciotta et al., 2011). The current output from 50 mg cell dry weight of *Anabaena variabilis* M-2 was completely inhibited by 0.1 M CCCP (Tanaka et al., 1988) and also stepwise addition of 5–200 μM CCCP to a *Lyngbya* biofilm let to decreasing photo response (Pisciotta et al., 2010). Twenty five μM CCCP had a reversible inhibitory effect on *Lyngbya* sp. and *Nostoc* sp. biofilms but addition of another 75 μM led to complete loss of photo response (Pisciotta et al., 2011).

##### *N-N’*-dicyclohexylcarbodiimide

*N-N’*-dicyclohexylcarbodiimide (DCCD) interferes with ATP synthesis by inactivating the ATP synthase (Yagishita et al., 1993).

##### Phenylmercuric acetate

Phenylmercuric acetate (PMA) interferes with the Q-cycle where electrons leaving PSI are fed back into the PQ pool, and was shown to decrease photo power outputs of *Nostoc* and *Lyngbya* biofilms (Pisciotta et al., 2010). When compared with CCCP, DCMU, DBMIB and DCCD, PMA was the only inhibitor that affected dark current generation from *Synechococcus* sp. (UTEX 2380) in the presence of HNQ (Yagishita et al., 1993).

##### Inhibitors of the respiratory terminal oxidases

All three respiratory terminal oxidases ARTO, COX and Cyd are blocked by potassium cyanide (KCN). Azide targets ARTO and COX. Both KCN and azide where demonstrated to act in a dose-dependent fashion but with possibly gradually arising toxic effects especially on *Nostoc sp.* biofilms at higher dosing (Pisciotta et al., 2011). Cyd can be selectively targeted by pentachlorophenol (PCP) with immediate effect on biofilms from *Lyngbya sp.* and *Nostoc sp.* (Pisciotta et al., 2011). Twenty five μM PCP reduced the exoelectrogenic activity that was completely inhibited when dosing another 75 μM.

#### Growth Mode and Biomass Composition

When planktonic cells (and a mediator) are employed, the volume of the electrolyte plays a key role for current output. Biofilm systems, on the other hand, rather depend on a large (three dimensional) surface area of the working electrode (Zou et al., 2009).

The establishment of a heterogeneous community consisting of photoautotrophic as well as heterotrophic microorganisms is considered to be an advantage for current output because on the one hand the overall (biofilm) stability is improved in a microbial community, and on the other hand it is assumed that photoautotrophs will provide organic matter as feedstock for heterotrophs, which in turn release electrons from the oxidation of these compounds to an anode (Rosenbaum et al., 2010). But as explained above, this will come at a significant loss in photon efficiency, so while this is a positive trait in early research development stages, an application of such a system would always be inferior in terms of the maximum achievable photon efficiency.

In terms of power output and efficiency, biofilms are not necessarily superior to planktonic cells (Table 1). High performances are rather a matter of the overall reaction environment, which includes the electrode materials and spacing, as well as supplements in the electrolyte. The choice between mixed species- or pure culture biofilms and planktonic cultures should depend on the actual research question and the envisaged length of the experiment. Biofilm experiments will always take more time for preparation since the biofilm has to be established first. Additionally, this growth form might hinder further analytics since extracellular polymeric substances have to be removed and representative sampling might be difficult due to heterogeneity of the biofilm. Biofilms might also not be suitable for inhibitor studies since homogenous distribution of respective molecules in a mature biofilm is unlikely. On the other hand, biofilm studies show more stable and prolonged power outputs (Zou et al., 2009) and do not rely on externally added redox mediators to support electron exchange between electrodes and microorganisms. A phototrophic consortium collected from a freshwater pond showed the highest electrogenic yields when compared to several pure culture cyanobacteria (Pisciotta et al., 2010).

The most commonly used procedure to grow a pure culture cyanobacterial biofilm on an electrode is to concentrate a liquid culture of known Chlorophyll *a* concentration by centrifugation, resuspension of the biomass in fresh medium and inoculation of the electrode with this cell suspension. Settling of cells and formation of biofilms should be allowed for several hours or up to few days before the measurement to ensure stabilization (Bombelli et al., 2012).

Besides biofilm and planktonic growth mode, there is another intermediate state when planktonic cells are concentrated via centrifugation and applied onto the anode as thick cell paste (Cereda et al., 2014). This quite artificial system has the advantage that a high and defined cell number can be applied directly on an electrode omitting a time-consuming biofilm growth step. The technique is also advantageous in case of non- or poor biofilm forming microorganisms like the model cyanobacterium *Synechocystis*.

#### Biomass

Quantifying growth and cell concentration is an essential first step to characterize the cellular fitness and to normalize electron output in a BPV. Quantifying biomass in planktonic systems seems straight forward (Mccormick et al., 2015) using, for instance, optical density (OD) measurements with a spectrophotometer or a turbidity probe. However, phototrophic cells are rich in pigments with strong light absorbance (Kopecna et al., 2012). In addition, the cell size and absorption properties can also change dynamically due to the accumulation/depletion of intracellular storage compound pools such as glycogen. It was demonstrated that a higher OD reading may just be a result of cell size change but an increase in cell number in later stages of growth experiments (David et al., 2018). These intrinsic properties of phototrophs could result in non-negligible and dynamic errors over the course of an experiment, when only using the OD as a proxy of biomass concentration (Myers et al., 2013). Ideally, a combination of OD measurement and other techniques, such as cell counting and fluorescence microscopy, should be employed.

In the case of a biofilm-based BPV, quantifying biomass is much more difficult. Methods to study the biofilm physiology and structure were comprehensively and critically reviewed by Azeredo et al. (2017). Here, optical methods are mostly applied for indirect measurement to correlate optical signals to biomass properties, but it is difficult to exclude background noise (Peeters et al., 2008). In order to achieve precise measurements, mostly invasive techniques are necessary that will disrupt the biofilm and render it useless for further characterization (Mccormick et al., 2011; Wenzel et al., 2018). Nevertheless, these methods may give much less errors in biomass quantification for BPV systems compared to heterotrophic BES systems, considering (i) the slow growth rate of cyanobacteria (Lopo et al., 2012; Zavřel et al., 2015a) and (ii) the currently reported short-batch time for BPV reactors ranging from minutes to hours (Saper et al., 2018; Zhang et al., 2018). The transparent or open designs of the BPV reactor (Pinhassi et al., 2016; Sawa et al., 2017) also provide the benefit of accessibility to *in situ* monitoring the biofilm using microscopy-based techniques.

#### Carbon Metabolism

Apart from the photosystems, changes in carbon metabolism caused by the anode might play an important role in BPV processes. Depending on how the electrons are transported to the anode, the anode can compete with the carbon assimilation for photo-electrons, or can even improve the carbon depletion process especially for the case of using hydrophilic mediators (Bombelli et al., 2011; Bradley et al., 2013).

Glycogen is the major storage carbohydrate for cyanobacteria. It is not only used as energy source during dark metabolism, but also functions as a dynamic cellular buffer system for electrons and energy (Zilliges, 2014; Cano et al., 2018). The change of the glycogen pool can give a direct indication of the electron flows in and out of carbon metabolism. An immunofluorescence based *in vivo* method was developed for visualizing the glycogen pool in the cells by tagging a fluorescence probe to glycogen (Skurat et al., 2017). This can possibly be applied for a continuous BPV batch equipped with fluorescence microscopy, but its precision is limited by quantitatively correlating the fluorescence signals to the glycogen amount. Mostly, the glycogen content is determined by using invasive enzymatic assays with the extractions of glycogen from the cells (Hasunuma et al., 2013; De Porcellinis et al., 2017).

The carbon metabolism may also be redirected into specific pathways by the electrode. This has been observed for many heterotroph-based bioelectrochemical processes (Kracke et al., 2016; Vassilev et al., 2018), but it is not known yet for cyanobacteria. Mapping the intracellular carbon metabolism could provide more insight into the interaction between carbon metabolism and electron flow toward the anode. Isotope labeled metabolic flux analysis has been successfully used for characterizing the carbon flux of cyanobacteria under different conditions (Knoop et al., 2013; You et al., 2014, 2015) and could in the future help to analyze the metabolic phenotype during BPV operation. This, however, still requires at least metabolic steady-state, which most likely could be achieved in planktonic systems rather than undefined biofilms. The development of quantitative metabolomics (Schwarz et al., 2013) and genome scale models (Montagud et al., 2010; Gopalakrishnan et al., 2018) for cyanobacteria will support such flux analysis efforts.

## Conclusion and Outlook

The study of BPVs is still in its infancy. It is difficult to compare different studies, as standardization on the biomass generation, growth method and the systems set-up is lacking. Observed currents described to date are orders of magnitude lower than traditional microbial fuel cells. A significant increase is necessary in order to make future applications of such systems a sensible endeavor. But there are encouraging studies that lay the basis for research into BPVs, generating highly relevant fundamental questions about electron transfer, which can be studied in BPV.

Current output is one limitation, but the dependence on artificial light sources and constant illumination make the application in realistic natural settings questionable. The influence of the heterogeneity of light and temperature (day-night, seasonal and intraday changes) will be important research questions, once current output under defined laboratory conditions can be significantly enhanced.

A fundamental understanding and targeted optimization of the microbes might help to achieve higher power outputs. Possible solutions in the future might include synthetic biology approaches to improve the electron transfer efficiency, such as introducing alternative electron transfer routes (Sekar et al., 2016; Schuergers et al., 2017). Targeted optimization, however, will require more standardized reporting of the data and fundamental understanding of the cellular physiology in BPV systems using systems biology approaches.

## Author Contributions

JT, BL, and JK designed the study. JT and BL drafted the manuscript and performed calculations for the table. BL prepared the figures. JK edited the final draft. All authors approved the final version.

## Conflict of Interest Statement

The authors declare that the research was conducted in the absence of any commercial or financial relationships that could be construed as a potential conflict of interest.
